# Ethogram of *Conepatus chinga* (Carnivora: Mephitidae) in Captivity: Approach to a Little-Studied Species

**DOI:** 10.3390/ani16091375

**Published:** 2026-04-30

**Authors:** Camila Oyanedel, Gabriel Perez, Diego Ramírez-Alvarez, Simón Cox, María José Ubilla, Gemma Rojo

**Affiliations:** 1 Escuela de Medicina Veterinaria, Facultad de Ciencias de la Vida, Universidad Andrés Bello, Santiago 8320000, Chile; c.oyanedelparra@uandresbello.edu (C.O.); g.prezabarca@uandresbello.edu (G.P.); 2 Unidad de Vida Silvestre, Servicio Agrícola y Ganadero, Rancagua 2820000, Chile; diego.ramirez@sag.gob.cl; 3 Instituto de Ciencias Agroalimentarias, Animales y Ambientales (ICA3), Universidad de O’Higgins, San Fernando 3070000, Chile; simon.cox@uoh.cl; 4 Facultad de Medicina Veterinaria y Agronomía, Universidad de Las Américas, Providencia 7500000, Chile; mubillac@udla.cl; 5 FaunaLab, Instituto de Ciencias Agroalimentarias, Animales y Ambientales (ICA3), Universidad de O’Higgins, San Fernando 3070000, Chile

**Keywords:** *Conepatus chinga*, ethogram, wildlife rehabilitation, camera trapping, environmental enrichment

## Abstract

We developed an ethogram of *Conepatus chinga* (South American hog-nosed skunk, chingue) from the continuous monitoring of a captive individual, with environmental enrichment, at a wildlife rehabilitation center in the O’Higgins Region, Chile. Using video camera traps, 17 days (408 h) of its activity were recorded, allowing us to identify daily activity patterns and times of exploration, locomotion, feeding, shelter use, and other relevant behaviors. The individual displayed a constant nocturnal activity pattern, starting around 9:00 P.M. and finishing at 5:00 A.M. The most frequent behaviors were walking and sniffing, evidencing active exploration guided by smell. No hunting/scavenging attempts were observed under these husbandry conditions. The primary refuge used was a small tunnel (approximately 30 cm deep) excavated under a wooden pallet covered with tree branches, which is consistent with refuges reported in wildlife. No visible defecation, urination, or musk release was recorded in the camera-trap footage. Given the limited observation coverage, we cannot determine whether these behaviors did not occur or occurred outside the recorded field of view. This study provides behavioral information and a descriptive baseline for this individual, including a pilot ethogram useful for research and captive management.

## Simple Summary

Wildlife rehabilitation centers often care for little-studied species, so it can be difficult to judge how an animal is doing and whether its enclosure meets its needs. We built a simple “behavior dictionary”, which is a list of observable behaviors, for the South American hog-nosed skunk (*Conepatus chinga*) by recording one adult male during initial health monitoring in a rehabilitation center in central Chile. Motion-activated video cameras captured 17 days of footage (408 h) while the enclosure included hiding places and shallowly buried food to encourage searching and digging. The individual followed a strong nighttime routine, becoming active around 9:00 in the evening and settling by about 5:00 in the morning. Most activity involved walking and sniffing, often followed by digging to uncover food; it drank regularly and mostly ate cat food pellets and insect larvae, ignoring other offered items. The animal preferred a short tunnel it dug under a pallet as its main refuge, and we did not observe scent spraying or visible urination or defecation. These observations provide a preliminary descriptive reference from a single rehabilitated individual, which may be useful for informing future, more systematic studies.

## 1. Introduction

Ethograms are standardized catalogs of species-typical behaviors, offering a practical backbone for evidence-based decisions in wildlife care because they turn observations into comparable, repeatable data [1]. In captive and managed settings, behavior time budgets and the detection of abnormal or stress-linked behaviors are widely used as animal-based indicators within welfare assessment frameworks, and they are especially useful for evaluating whether environmental enrichment is improving welfare over time [2]. Remote video methods, such as camera traps, can strengthen this approach by reducing observer disturbance and providing continuous coverage that captures nocturnal or cryptic behaviors that are easy to miss during direct observation [3].

Carnivores are a high-priority group for ethogram-based monitoring because captivity can constrain key natural behaviors such as ranging, foraging, and digging. Wide-ranging carnivores are prone to welfare challenges that often manifest behaviorally. Despite the growing zoological and welfare literature on behavioral monitoring, ethograms remain uneven across carnivore lineages and contexts [4]. For Mephitidae, published ethogram-style work is still comparatively sparse; a clear example is the video camera documentation of previously undescribed scent-marking behaviors in striped skunks (*Mephitis mephitis*), which illustrates both the value of camera-based observation and the opportunity to expand standardized behavioral baselines in skunks [5].

The South American hog-nosed skunk, or chingue (*Conepatus chinga*, Molina, 1782), is a primarily nocturnal South American mephitid common in human-modified landscapes, and detectable with camera traps. Like other mephitids, it has a pair of anal scent glands that secrete a noxious fluid for defense [6]. Ecologically, *C. chinga* is an omnivorous opportunistic forager, primarily consuming arthropods, eggs of ground-nesting birds, geophyte bulbs, and occasionally micro-mammals and amphibians [7,8,9], and demonstrating flexible use of habitats, traits that are directly relevant to designing enrichment that promotes naturalistic digging/foraging and to interpreting activity patterns under temporary holding conditions. However, most published information is framed around field ecology (diet, habitat use, activity rhythms) [10,11], rather than a standardized ethogram tailored to rehabilitation settings [12,13].

We developed a pilot ethogram for a single *C. chinga* during a short-term holding/observation period prior to release, using continuous remote camera monitoring paired with basic enrichment. Specifically, we (i) defined a species- and context-appropriate set of behavioral categories and explicit scoring rules for each behavior (i.e., an operational definition to ensure consistent classification from video), (ii) quantified an activity budget and selected behavioral metrics during short-term captive holding, and (iii) used these metrics to describe enrichment interactions and to note potential welfare-relevant patterns (e.g., prolonged inactivity or repetitive behaviors) that could inform husbandry adjustments in similar holding contexts. This initial approach seeks to provide a baseline characterization of the animal’s behavioral patterns, guide future ethological studies, and serve as a reference for management and conservation plans for the species.

## 2. Materials and Methods

### 2.1. Study Site and Subject

This study was conducted at the Hacienda Cauquenes Wildlife Rehabilitation Center, in the municipality of Requínoa, O’Higgins Region, Chile (344489mE/6210433mS H19 UTM). The adult male *C. chinga* arrived at the establishment on 17 November 2024, because it was found outside its habitat in a garden in the town of Lolol (257763mE/6157785mS H19 UTM). After veterinary clinical evaluation, it was found to be healthy and in adequate nutritional condition (body condition score 3/5) with no injuries or signs of disease. It was kept in the enclosure only for temporary holding before being reintroduced to the wild.

### 2.2. Feeding and Enrichment Protocol

**Figure 1:**
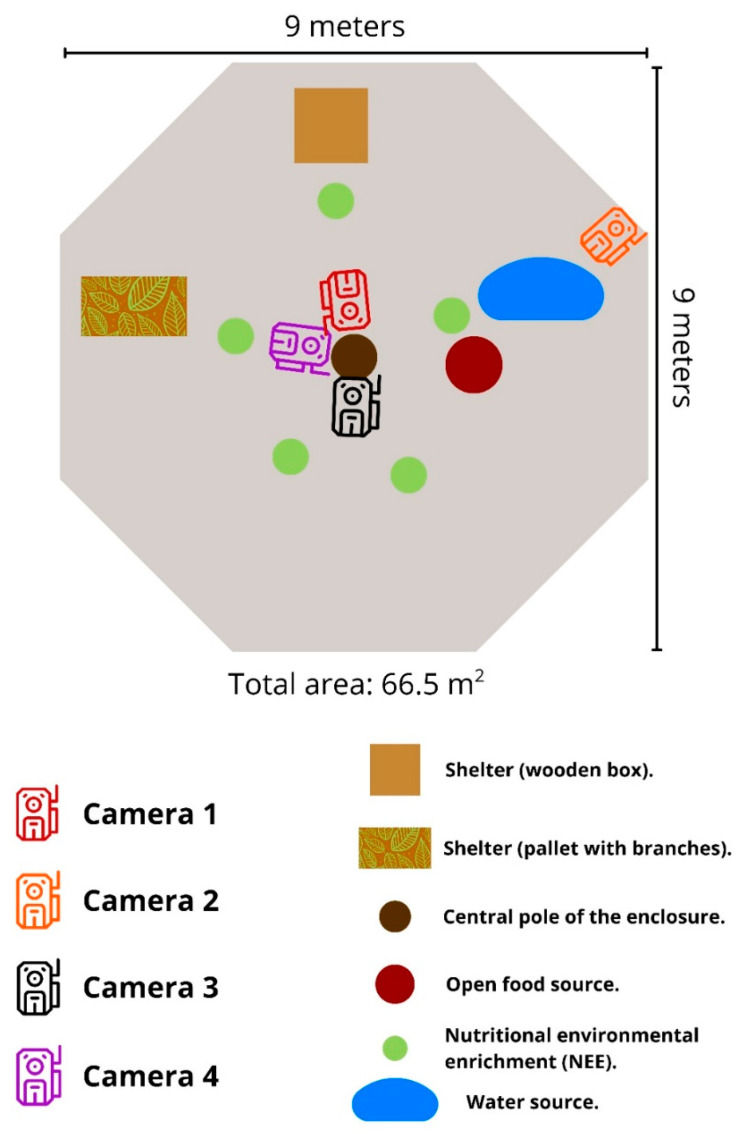
Layout of the enclosure, arrangement of cameras, and environmental enrichment elements.

The enclosure (Figure 1) was a 9 m wide by 9 m long octagon, with walls built of structural wood on concrete pillars, a metal mesh roof, and an exterior perimeter made of metal mesh. It had natural flooring and structures that facilitated spontaneous behavior. During its stay, occupational, physical, and nutritional enrichment conditions were provided, designed to promote natural behaviors and the animal’s well-being (Figure 1): It was provided two shelters, one a wooden box and the other a wooden pallet covered with tree branches; an open water source (pond); an open food source; and many pieces of nutritional environmental enrichment (NEE), consisting of cardboard boxes containing cat food, insect larvae (*Tenebrio molitor*), and, in some cases, chives and chicken eggs. Food was available ad libitum, and all boxes were tied tightly and buried shallowly (3–10 cm deep) with a garden spade. All bury sites were inspected daily to check for feces or urine.

### 2.3. Data Collection

Data were collected by installing four camera traps in different areas of the enclosure to capture the widest range of behaviors. The cameras were active continuously—from 25 November 2024 to 4 December 2024, and from 5 December 2024 to 17 December 2024—with 17 days (408 h) of effective day and night monitoring, which covered the individual’s activity cycle during pre-release observation.

We used Bushnell 24MP Trophy Cam, model 119719CW (Bushnell Corporation, Pittsburgh, PA, USA), with the following settings: Mode: Video; Sensor level: Auto; Camera mode: 24 h. The cameras were disposed as follows (Figure 1):

Camera 1: Directed toward the wooden box provided as a refuge, it captured behaviors associated with resting and hiding. Also with pieces of NEE to capture foraging behaviors. It operated with a 10 min delay.

Camera 2: Directed to the main water source, open food source, and NEE pieces to stimulate foraging. It had a 1 min delay, allowing for greater temporal resolution to detect interactions with the resources.

Camera 3: Directed to an open area of the enclosure not recorded by the other cameras, to monitor general movements. Also with pieces of NEE to capture foraging behaviors. It had a 10 min recording interval.

Camera 4: Directed to the wooden pallet covered with tree branches, where the animal dug a short tunnel (approximately 30 cm deep) as its principal refuge. Also with pieces of NEE to capture foraging behaviors. This camera operated with a 1 min delay to record its foraging and exploration behavior in more detail.

### 2.4. Behavioral Classification

The ethogram was constructed based on a reference for mustelids [14], complementing it with the behaviors that we observed in our study, adjusting and defining a behavioral repertoire specific to the captive animal (Table 1).

### 2.5. Data Analysis

For data analysis, video recordings were reviewed, recording the time (in seconds) spent on each behavior in the selected repertoire in a structured spreadsheet. Behavior frequencies and durations were analyzed quantitatively using R software version 4.4.3 [15]; processing and quantitative analysis of behavior frequencies and durations were performed with functions from the dplyr package, such as group_by() for grouping, summarise() for summarizing, and proportion calculations, and the ggplot2 package for data visualization. Data were processed to group the recorded behaviors by day and by full-hour intervals. This allowed for a temporal breakdown of the activity patterns. Subsequently, the percentage expression of each behavior was calculated, both per camera and across different time periods. To visualize the temporal distribution of activity, bar plots were created showing the percentage of video records per hour, which facilitated the identification of the individual’s circadian rhythm. This analysis identified the hours with the highest activity and the most frequent behaviors throughout the day.

## 3. Results

**Figure 2:**
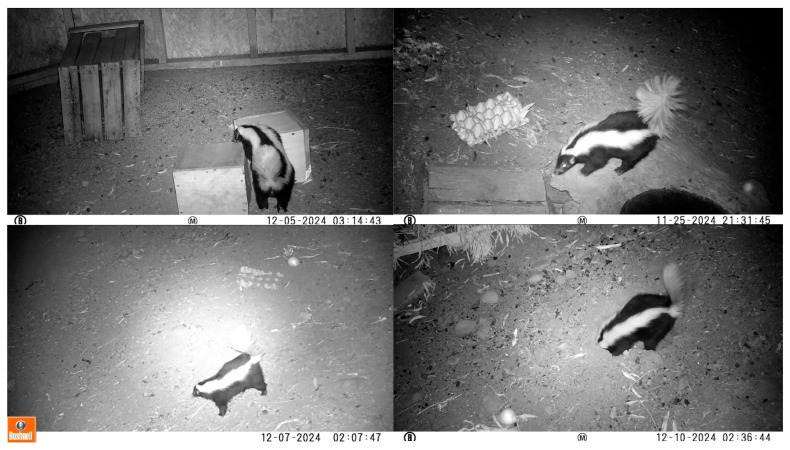
Different views of *C. chinga* as captured by cameras 1, 2, 3, and 4, respectively.

The four cameras showed a different composition of behaviors, as they had different monitoring approaches (Figure 2).

**Figure 3:**
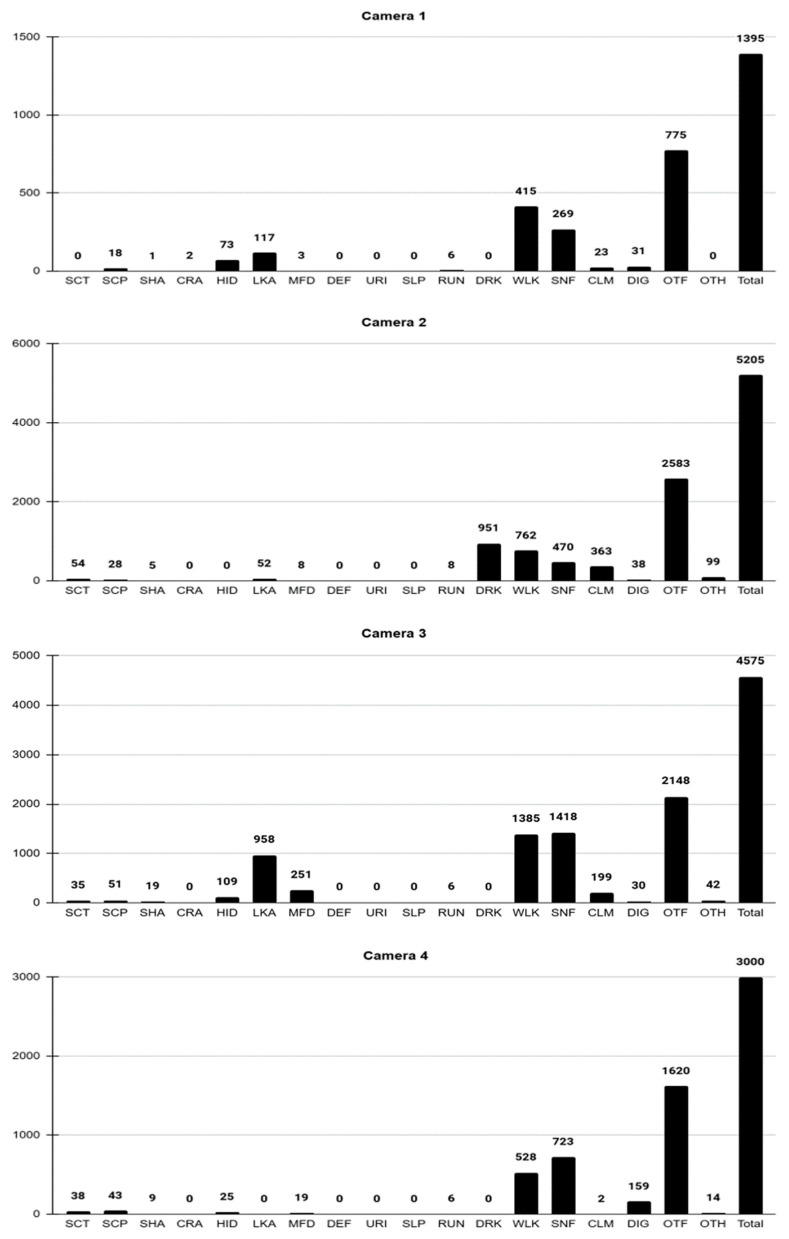
Behaviors under evaluation, expressed in seconds (s), for each camera.

The time spent on the different behaviors under evaluation, expressed in seconds (s), for each camera, can be seen graphed in Figure 3.

Camera 1:

Activity of the individual was recorded for 1395 s. Of this, 775 s corresponds to activations in which the specimen was not visible in the frame (OTF), due to its constant exploratory behavior, roaming the enclosure and generating multiple activations from different locations.

WLK and SNF behaviors stand out, lasting 415 s and 269 s, respectively, agreeing with the places where NEE was available. DIG was demonstrated for 31 s, finding NEE pieces, HID for 73 s in the wooden box, and SCP for 18 s, usually after SNF.

Camera 2:

A total of 5205 s of activity was recorded. OTF (2583 s), WLK (762 s), and SNF (470 s) were, again, notable.

Due to the presence of the water fountain in this approach, DRK behavior was expressed for 951 s. This approach also included NEE, which increased olfactory behavior compared to camera 1. CLM occurred for 363 s, using logs provided as physical environmental enrichment, and OTH behavior was expressed for 99 s: stumbling, falling, and swinging.

Camera 3:

A total of 4575 s of activity was recorded, highlighting the expression of OTF (2148 s), SNF (1418 s), WLK (1385 s), and LKA (958 s) through continuous exploratory behavior in NEE places.

DIG behavior was displayed for 30 s in areas with NEE. Due to the animal’s great digging ability, it did not take long for it to unearth the items. After unearthing them, it manipulated them (MFD) for 251 s, eating only the cat food pellets and insect larvae without touching chives or chicken eggs.

Behaviors such as SCT and SCP were displayed for 36 and 51 s, respectively, after digging and sniffing. HID was demonstrated for 109 s, and OTH (42 s) was expressed: stumbling and rocking.

Camera 4:

A total of 3000 s of activity was recorded, with 1620 s of OTF. This area included the small tunnel under a wooden pallet covered with tree branches, which the animal used as its primary refuge. Most of the observed activity was concentrated outside the pallet, where the individual frequently displayed exploratory behaviors.

Of note was DIG behavior, recorded for 159 s, directly associated with the search for NEE pieces. SNF was recorded for 723 s, and WLK for 528 s, reflecting active exploration directed toward the NEE pieces.

Also observed were RUN (6 s), MFD (19 s), mainly when accessing unearthed NEE pieces, and HID (25 s), a behavior that occurred when the individual entered the space under the pallet after periods of activity. SCT and SCP were observed for 38 and 43 s, respectively.

**Figure 4:**
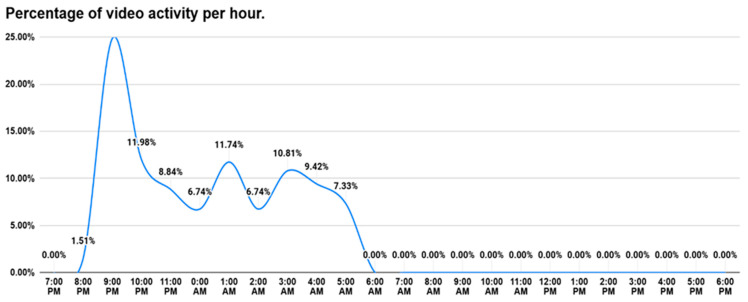
Percentage of videos recorded by hour.

The hour with the highest percentage of recordings was 9:00 P.M., representing 24.88% of the total videos captured. This was followed by 1:00 A.M. with 11.74%, and 10:00 P.M. with 11.98%, reinforcing the nocturnal pattern of activity already observed in the qualitative analysis. In contrast, between 6:00 A.M. and 8:00 P.M., activity was practically nonexistent, with 0% recorded at 6:00 AM and only 1.51% at 8:00 P.M. (Figure 4).

During the observation period, the individual showed a clear dietary preference for cat food pellets (97%) and insect larvae (*T. molitor*) (3%). No ingestion of chives (*Allium schoenoprasum*), chicken (*Gallus gallus*) eggs, or a dead European rabbit (*Oryctolagus cuniculus*) offered as carrion or attempts to hunt a wild rodent (*Rattus rattus*) that spontaneously entered the enclosure were recorded.

## 4. Discussion

### 4.1. Activity Patterns

*C. chinga* displays a clearly defined activity pattern in captivity (9:00 P.M.–5:00 A.M.). This pattern remained constant throughout the observation period, with minimal variations of a few minutes between days. This regularity suggests a well-established nocturnal circadian rhythm, consistent with previous reports for the species [16,17] and for other members of the genus *Conepatus* [18].

The greatest activity was concentrated at 9:00 PM, coinciding with the moment the individual awakened and began actively exploring its enclosure in search of food and water. This initial phase of exploration was consistent and marked the beginning of its nocturnal activity period. The only exception to this pattern occurred on the day the individual was introduced to the enclosure, at which time it displayed both daytime and nighttime activity, possibly associated with the initial exploration of the new environment.

### 4.2. Exploration

Within the behavioral repertoire observed in the individual, exploratory behaviors, especially walking and sniffing, were the most frequent during the monitoring period. These behaviors were present in all recordings and reflect the animal’s clear willingness to interact with its physical and sensory environment. Active exploration [19,20] and behavioral innovation to manipulate objects and solve tasks [21,22] are expected characteristics in members of the Mephitidae family.

The observed exploratory behavior was predominantly mediated using smell, especially in areas of the enclosure where resources such as food or shelter were concentrated. This trend coincides with previously described behavior; *C. chinga* selects habitats based on prey availability, reflecting exploration oriented toward the detection of and active search for food [23]. In our study, the individual was observed to intensively use its olfactory capacity to locate resources, even to the detriment of its other senses: on multiple occasions, tripping events increased during sniffing bouts, suggesting reduced attention to uneven terrain while investigating olfactory cues, as has been described in other members of the group [24,25].

Additionally, other exploratory behaviors, such as climbing and digging, were recorded, although less frequently. These actions complement the individual’s exploratory profile and demonstrate its ability to exploit structural resources and substrates within the enclosure.

*C. chinga* interaction with environmental enrichment was active and sustained. Both nutritional enrichment, through the search for and detection of buried or hidden food, and occupational enrichment, through structural elements such as shelters, logs, or pallets, were regularly used. The individual not only managed to find hidden food but also manipulated and opened structures designed as simple cognitive challenges, indicating a good level of motivation and adaptive response to the environment.

The frequent display of exploratory behaviors observed in this work allows us not only to assess the well-being of individuals under human management, but also to establish baseline behavioral parameters that can be used as a reference in rehabilitation plans, pre-release programs, and future research in the wild. Understanding how the species interacts with its environment, especially through olfactory exploration and active resource foraging, is essential for designing more informed conservation strategies tailored to its ecological and behavioral needs [26,27].

### 4.3. Locomotion

The individual’s locomotor repertoire was dominated by walking, climbing, and running. Walking was the primary mode of movement and was characterized by being slow and guided using smell. This mode of locomotion, with the head constantly close to the substrate, resulted in tripping over objects in the environment on several occasions, suggesting a limited visual capacity to detect obstacles while exploring.

*C. chinga* does not rely on speed to capture prey or escape predators; on the contrary, its potent defense mechanism, through the secretion of musk, tends to deter potential predators such as foxes and pumas, which generally avoid contact with these animals [16,28]. This combination of slow locomotion and chemical defense allows it to maintain a low-risk, exploratory lifestyle. Consistent with this, in our monitoring, running behavior was almost absent (only 6 s recorded in the entire observation period), while walking dominated the locomotor repertoire, reinforcing the interpretation that the species favors a low-risk exploratory strategy supported by chemical defense rather than speed-based escape.

Climbing was a significant behavior within the category, observed primarily in elevated areas of the enclosure, such as the watering area, logs, and shelter structures. In these cases, the behavior was closely related to the search for resources, especially food and water, demonstrating an active willingness to explore different levels of the available space.

Running behavior was rarely recorded, consistent with the absence of threats or disruptive stimuli in the environment. During some quiet/resting periods, mild body swaying was observed. Because this behavior is non-specific and can be influenced by multiple factors (e.g., posture during rest, drowsiness, stress, or individual variation), and although there are previous descriptions of low visual acuity in Mephitidae [24,25], we do not infer visual impairment or other clinical conditions from this observation.

### 4.4. Nutrition and Hydration

The species, although belonging to the order Carnivora, exhibits omnivorous feeding habits in nature, with a diet preferentially focused on insects and other invertebrates found during its active foraging at ground level [11]. In our records, feeding behaviors showed a clear and repeated pattern of sniffing, digging, and manipulating food, which evidences a search strategy based predominantly on the use of smell, accompanied by the effective use of the animal’s powerful claws to access buried resources.

Under the captive diet provided, the individual consumed primarily commercial cat food pellets, with occasional consumption of insect larvae. Pellets were used as a simple enrichment by burying them; however, they were continuously available within the enclosure. Other items offered (e.g., chives, chicken eggs, and a European rabbit carcass) were not consumed; the carcass was briefly investigated by olfaction but not otherwise manipulated. A wild rat entered the enclosure and remained for several days; no approach or capture attempts were observed. Since the feeding conditions were artificial and the individual had continuous access to highly palatable pellets, this likely influenced motivation and foraging behavior (including satiety effects), potentially suppressing investigation of alternative foods and reducing incentive to interact with carrion or pursue live prey. Accordingly, we present these findings as context-specific observations rather than evidence of species-level dietary preference, scavenging behavior, or hunting tendency, as has been reported in previous studies on its trophic ecology [10,11].

The recorded behavioral sequence of sniffing, digging, and food manipulation was consistently observed across different locations within the enclosure, suggesting that these actions are a core part of the animal’s natural foraging repertoire. Furthermore, the individual consistently located and unearthed buried pellets, indicating active olfactory-guided searching and digging behavior under the enrichment protocol.

Across the monitoring period, we recorded an average of 11 drinking events per day, based on camera-trap detections at the water source. In our case, because the animal’s main food intake was dry extruded cat food pellets, we estimate that its water requirement was greater than in the wild, where this behavior will be determined by the liquid content of the prey it consumes [29,30].

### 4.5. Shelter

During the entire monitoring period, the individual used as its primary shelter a short tunnel approximately 30 cm deep, which it dug on its first night entering the enclosure, under a wooden pallet covered with tree branches. This structure provided a closed and protected space and was preferred over other shelter types, such as smaller wooden boxes, which it used only temporarily. This is consistent with the shelters the species uses in the wild, selecting cavities in the ground, spaces between dense vegetation, or areas under branches and plant debris as resting and protective sites [31,32].

This pattern suggests that the individual used the shelter not only for resting, but also for protection, consistent with its cryptic biology and nocturnal habits. The shelter acted as a return point after active periods, which aligns with territorial strategies observed in other solitary carnivores [33,34,35].

### 4.6. Excretion and Chemical Defense (Feces, Urine, Musk)

During the monitoring period, no behavior instances of the individual urinating, defecating, or releasing musk were recorded. We also did not find physical evidence of biological waste excretion during active inspections of the enclosure after the animal’s release.

Some studies describe certain species of animals rolling or self-anointing in their feces, thus blending them into the soil and fur [36,37]. In our case, since the cameras did not allow us to see what was happening inside its shelter tunnel under the branch-lined pallet, we cannot rule out that the specimen performed this behavior in this location, blending the excreted material. This could explain the absence of visible feces in the enclosure. However, for *C. chinga*, there are records of finding feces in their normal form in the wild [11,38].

Another hypothesis, more consistent with the potentially stressful conditions in captivity, is that the individual could have buried its feces, a behavior observed in numerous species as a strategy to avoid leaving traces detectable by predators [39,40,41]. In some of our visual recordings, we can see how, after remaining crouched for a few seconds, the individual makes movements with its forelimbs, throwing soil backward, similar to the behavior of cats covering their feces in a litter box. Although we were unable to clearly identify this action as excretory and burying behavior, it could explain the absence of feces in the enclosure.

### 4.7. Grooming

No direct grooming behaviors were recorded, involving the use of the limbs, the mouth, or rubbing against other objects. The behaviors recorded within the categories of “scratching with teeth” or “scratching with paws” mostly occurred after active exploration or feeding, and could be interpreted as removing dirt or other debris from the face or body rather than grooming behaviors per se.

No behaviors associated with body bathing were recorded either. The camera monitoring the water source (the only area with water) showed that the individual only lightly submerged its limbs in the water, without submerging the rest of its body or performing actions that could be interpreted as bathing. We did not find any descriptions of water bathing in the wild in the literature available for this species, so it appears to be refractory to this element beyond drinking it.

### 4.8. Limitations of the Study

This study is limited by the fact that observations were conducted on a single captive individual. Therefore, the results cannot be generalized to the entire species and should be interpreted as part of an exploratory approach rather than definitive patterns of behavior. Nevertheless, the findings provide valuable baseline information and highlight the importance of conducting future research with larger sample sizes, multiple individuals, and, ideally, free-ranging animals to confirm and expand upon these preliminary insights.

## 5. Conclusions

This study provides the first characterization of the behavior of *C. chinga* in captivity with environmental enrichment. This is the first structured ethogram report for this species in a Chilean rehabilitation context, demonstrating feasibility of continuous camera-trap ethogram capture during pre-release observation. Finally, this report illustrates how a simple ethological framework and remote video monitoring can be used to produce a preliminary, context-specific description of behavior for a little-studied species during short-term captive holding. Given the single-individual design and artificial diet, these observations should be interpreted cautiously and are intended primarily to inform future, more systematic ethological studies and to support husbandry monitoring in comparable holding contexts.

## Figures and Tables

**Table 1:** Ethogram of *C. chinga* in captivity.

Initials	Behavior	Operational Description
SCT	Scratching with teeth	Animal brings its mouth into contact with a specific body region and repeatedly closes the jaws or scrapes the incisors/canines against the skin or fur, producing short, repetitive biting or nibbling movements.
SCP	Scratching with paws	Animal raises one or both fore- or hindlimbs and rubs them repeatedly against a specific body region (e.g., ear, flank, neck), producing rapid, back-and-forth movements with the digits or claws in direct contact with the skin or fur.
SHA	Shaking	Animal performs a rapid, whole-body oscillation, starting with a lateral movement of the head and propagating along the trunk to the tail. This movement lasts less than 2 s and typically results in repositioning of the fur.
CRA	Crawling	Animal moves forward with the body maintained close to the substrate, keeping the abdomen in continuous contact.
HID	Hiding	Animal partially or completely enters a shelter or cover, and remains inside with reduced visibility of the body. Duration varies from brief (seconds) to prolonged (minutes or more).
LKA	Looking around	Animal pauses locomotion or other ongoing activity and orients the head and eyes toward the surroundings, often turning the head.
MFD	Manipulating food	Animal takes, moves, or explores food, followed by ingestion.
DEF	Defecating	Animal eliminates feces.
URI	Urinating	Animal eliminates urine, either through bladder emptying (a larger amount of urine) or through marking.
SLP	Sleeping	Animal rests lying down with its eyes closed, with no movement.
RUN	Running	Animal performs a rapid, sustained movement involving the coordinated extension and flexion of all four limbs, with the body typically raised and stretched out.
DRK	Drinking water	Animal ingesting water from the drinking fountain.
WLK	Walking	Animal moves from one direction to another, without rushing or moving extremely slowly, moving its limbs in a coordinated pattern.
SNF	Sniffing	Animal approaches with its nose to the ground or objects, inhaling rapidly, with active nasal movements.
CLM	Climbing	Animal climbs up vertical or sloping physical structures within the enclosure.
DIG	Digging	Animal uses its front legs to dig up soil or substrate.
TRP	Tripping	Animal trips, temporarily losing its balance or causing a fall.
STR	Stretching	Animal stretches its body and limbs, often after resting or sleeping.
SWY	Swaying	Animal exhibits a repetitive movement of its body, swaying it without moving.
JMP	Jumping	Animal propels its body upward using its hind limbs.
ENR	Interacts with enrichment	Animal explores or uses environmental enrichment elements (boxes, leaves, etc.), manipulating them with its hands, sniffing or biting them.
MSK	Musk release	Animal raises its tail and backs away, discharging its anal glands as a sign of avoidance or defense against a stimulus that causes it discomfort.
OTF	Out of frame	Animal is outside the camera frame, so it is not visible, but it can be inferred that it is active.
OTH	Other	The animal exhibits other behaviors not previously described.

## Data Availability

The original contributions presented in this study are included in the article. Further inquiries can be directed to the corresponding author.
